# Blood lipid profiles and peripheral blood mononuclear cell cholesterol metabolism gene expression in patients with and without methotrexate treatment

**DOI:** 10.1186/1741-7015-9-4

**Published:** 2011-01-13

**Authors:** Der-Yuan Chen, Hui-Min Chih, Joung-Liang Lan, Hsin-Yueh Chang, Wei-Wen Chen, En-Pei Isabel Chiang

**Affiliations:** 1 Department of Food Science & Biotechnology, National Chung Hsing University, Taichung, Taiwan

## Abstract

**Background:**

Methotrexate (MTX) is the most commonly prescribed disease-modifying antirheumatic drug (DMARD) in rheumatoid arthritis. ATP-binding cassette transporter-A1 (ABCA1) and 27-Hydroxylase (HY27) are known antiatherogenic proteins that promote cellular cholesterol efflux. In THP-1 macrophages, MTX can promote the reversal of cholesterol transport, limit foam cell formation and also reverse COX-2 inhibitor-mediated downregulation of ABCA1. Despite its antiatherogenic potential *in vitro*, the impact of clinical use of low-dose MTX on cholesterol metabolism in humans is unknown. Objective of the study was to examine whether clinical MTX use is associated with altered blood lipids and/or *ABCA1/HY27 *expressions.

**Methods:**

In all, 100 rheumatoid arthritis subjects were recruited from a medical center in central Taiwan. Plasma lipid profiles and peripheral blood mononuclear cell *HY27 *and *ABCA1 *expressions were compared between subjects taking MTX (MTX+) and other disease-modifying antirheumatic drugs (DMARDs) (MTX-). Dietary intake was assessed by a registered dietician.

**Results:**

Though no difference observed in the blood lipids between MTX+ and MTX- subjects, the expressions of *ABCA1 *and *HY27 *were significantly elevated in MTX+ subjects (n = 67) compared to MTX- subjects (n = 32, p < 0.05). ABCA expression correlated with MTX doses (r = 0.205, p = 0.042), and MTX+ subjects are more likely to have increased *HY27 *compared to MTX- subjects (OR = 2.5, p = 0.038). Prevalence of dyslipidemia and overweight, and dietary fat/cholesterol intake were lower than that of the age-matched population. Although no differences were observed in the blood lipids, the potential impacts of MTX on cholesterol metabolism should not be overlooked and the atheroprotective effects from MTX induced *HY27 *and *ABCA1 *expressions may still be present in those persons with pre-existing dyslipidemia.

**Conclusions:**

We demonstrated novel findings on the increased gene expressions of atheroprotective protein *HY27 *and *ABCA1 *in human peripheral blood mononuclear cells (PBMCs) with clinical use of low-dose MTX. Whether MTX induced *HY27 *and *ABCA1 *expressions can protect against cardiovascular disease in patients with chronic inflammation through the facilitation of cholesterol export remains to be established. Further studies on the impacts of low-dose MTX on hypercholesterolemic patients are underway.

## Background

Methotrexate (MTX) is the most commonly prescribed disease-modifying antirheumatic drug (DMARD) for human rheumatoid arthritis. MTX is a folate antagonist that inhibits dihydrofolate reductase activity and is used for its anti-inflammatory and immunosuppressive properties in rheumatoid arthritis [1]. MTX blocks a number of enzymes involved in purine and pyrimidine metabolism. MTX promotes adenosine release and adenosine, acting at its receptors, mediates the immunologic and anti-inflammatory effects of MTX in the treatment of rheumatoid arthritis [2-4].

Dyslipidemia is the elevation of plasma cholesterol, triglycerides, or a low/high density lipoprotein that usually results from excessive dietary intake of saturated fat, cholesterol, or trans fats. We recently discovered in a pilot study that some arthritis patients on low dose MTX had altered blood lipids compared to those not taking methotrexate [5]. We postulated that these altered blood lipid profile is related to the changes in ATP-binding cassette transporter A1 (ABCA1) and 27-hydroxylase (HY27), as a previous *in vitro *study reported that MTX induced *HY27 *expression and reversed cyclo-oxygenase 2 (COX-2) inhibitor-mediated downregulation of *ABCA1 *in a macrophage cell model.

The mitochondrial cytochrome P-450 sterol HY27 catalyzes the initial oxidation steps in the bile acid synthesis from cholesterol [6]; ABCA1 is a cell membrane-bound protein responsible for the secretion of excess cholesterol, phospholipids, and other lipophilic molecules across the plasma membrane from cells. Both HY27 and ABCA1 play crucial roles in cholesterol metabolism in most tissues of the body [7].

Atherosclerosis contributes to cardiovascular disease (CVD), morbidity, and mortality in autoimmune diseases, including: systemic lupus erythematosus and rheumatoid arthritis [8]. Impaired cholesterol homeostasis has been reported in patients with rheumatoid arthritis and lupus erythematosus [8-10]. Incubation of systemic lupus erythematosus in patients' plasma significantly decreased the expression of *HY27 *in THP-1 human monocytes and further increased the THP-1 macrophage foam cell transformation from monocytes [11]. These results imply a role of immune dysfunction in human atherogenesis, and certain immunological mediators may perturb the cholesterol homeostasis in human monocytes/macrophages and endothelium. By inhibiting the inflammatory response, MTX and other DMARDs may reduce the risk of CVD by decreasing the exposure of atherogenic mediators in these patients.

*In vitro *MTX treatment increases *HY27 *mRNA expression and blocks COX2 inhibitor-induced downregulation of *HY27 *in human THP-1 monocyte/macrophages [12]. In THP-1 macrophages MTX has been reported to promote the reversal of cholesterol transport through adenosine A2A receptor activation and limits foam cell formation [12]. In addition, MTX reverses COX-2 inhibitor-mediated downregulation of *ABCA1*, suggesting an atheroprotective effect of MTX [12]. We hypothesized that clinical use of low-dose MTX in humans can alter blood lipid metabolism via induction of *ABCA1 *and *27HY.*

The objective of the present study were to (1) compare the blood lipid profile as well as gene expression of these antiatherogenic proteins between subjects taking MTX (MTX+) and other DMARDs (MTX-), and (2) examine the associations among MTX dose, duration and lipid profiles, as well as the expression of *ABCA1 *and *HY27 *in the peripheral blood mononuclear cells (PBMCs) of humans with chronic inflammation.

## Methods

### Subjects

A total of 100 adults (aged above 18 years) with rheumatoid arthritis fulfilling the American College of Rheumatology 1987 revised criteria for rheumatoid arthritis [13] were recruited prospectively from the Allergy, Immunology and Rheumatology outpatient clinic at the Department of Immunology and Rheumatology, Taichung Veterans General Hospital, Taiwan between 2007 and 2009. The study protocol was approved by the clinical research ethics committee, and informed consent was obtained from each participant prior to enrollment. Subjects with pregnancy, anemia (hemoglobin 10 mg/dL or lower), thrombocytopenia (platelet count below 50,000 cells/μL), abnormal serum hepatic transaminase (aspartate aminotransferase or alanine aminotransferase above 50 IU/L), renal insufficiency (serum creatinine above 1.5 mg/dL), diabetes or cancer were excluded. None of the study subjects were using antihyperlipidemic drugs during the study period. Blood chemistry and hematology screening were performed to ensure eligibility before enrollment. All patients were taking medications for rheumatoid arthritis symptom relief, and therefore disease activity was controlled during the study period. No changes in medication had been made for 1 month prior to enrollment for any of the subjects. Levels of C-reactive protein were determined by enzyme immunoassay [14] (Virgo C-reactive protein 150 kit; Hemagen, Waltham, Massachusetts, USA).

### Human study experimental protocol

Subjects were asked to fast overnight for 12 h for the study blood draw. In the following morning, fasting blood samples from the subjects were collected into ethylenediaminetetraacetic acid (EDTA) tubes and chilled immediately on ice for the determination of total cholesterol (TC), triglyceride (TG), high-density lipoprotein-cholesterol (HDL-C), low-density lipoprotein cholesterol (LDL-C), and for RNA extraction from the PBMCs. Each patient was then instructed to complete the Stanford Health Assessment Questionnaire (HAQ) [15], which has 13 categories of questions that evaluate how well the patient is able to perform routine activities.

### Plasma lipid profiles in subjects taking MTX and other DMARDs

Plasma total lipid was determined enzymatically with an autoanalyzer (Hitachi 7070; Hitachi, Tokyo, Japan) using clinical chemical kits (Wako Co, Osaka, Japan). TC, TG, HDL-C, LDL-C were determined by Randox reagent kit (Randox Laboratories Ltd, Crumlin, UK).

### *ABCA1 *and *HY27 *expression in human PBMCs

**Figure 1:**
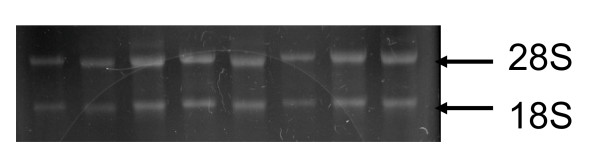
**The integrity and quality of all RNA samples were checked on agarose gels before being reverse transcribed**.

PBMC RNA was isolated using TRIzol (Invitrogen, Carlsbad, CA, USA) and dissolved in nuclease-free water. The quality of all RNA samples was checked on agarose gel (Figure 1) and the quantity of total RNA was measured by spectrophotometer. Complementary DNA (cDNA) was copied from 2 μg total RNA using Moloney murine leukemia virus reverse transcriptase (Promega, Madison, WI, USA) primed with oligo(dT). Equal amounts of cDNA were taken from each reverse transcription (RT) reaction mixture for real-time polymerase chain reaction (PCR) amplification [16], using cholesterol *HY27*-specific primers or *ABCA1*-specific primers a well as 18S as internal control primers. Real-time PCR analysis was performed using the SYBR Green PCR Reagents Kit (Applied Biosystems, Foster City, CA, USA).

### Dietary intake of study subjects

For assessment of dietary intake, subjects were instructed to complete a food record, and each subject was interviewed by a registered dietitian for validation of the food record [17] on the day of blood collection. A colored book with 1:1 size food photos were used to standardize portion size among subjects. Nutrient composition was calculated with the use of Nutritionist Professional software (E-kitchen Business Corporation, Taipei, Taiwan), in combination with the Taiwan food composition table, established by the Department of Health in Taiwan.

### Statistical analysis

Statistical analyses were performed using SYSTAT software (Version 11.0) for Windows (SYSTAT, Richmond, CA, USA). Demographic and hematological data were compared by Mann-Whitney U test between the two groups. For categorical variables, differences between two groups were assessed by χ^2 ^test. Pearson correlation coefficients were calculated between continuous variables including PBMC *ABCA1 *expression, *HY27 *expression, the MTX duration (months), and the MTX weekly dose (mg) in subjects taking low dose MTX. Odds ratios (ORs) with 95% confidence intervals (CI) for elevated (above the median) *ABCA1 *o*r HY27 *gene expression were calculated from unconditional logistic regression models according to the MTX use or not. Results were defined statistically significant at p <0.05.

## Results

### Demographic information

In all, 100 patients with rheumatoid arthritis were enrolled. One subject dropped out due to her concerns over a 15 mL blood draw for RNA extraction and lipid profiles in addition to the patient's routine monthly blood draw for blood chemistry. Characteristics of the 99 subjects who completed the study are shown in Table 1. In all, 67 patients (67%) used MTX, and 32 patients had never used MTX (n = 17, 17%) or discontinued use of MTX for at least 2 months due to side effects (n = 15, 15%). Other DMARDs used by these subjects included prednisolone (88 patients, mean weekly dose 39.4 mg), salazopyrin (84 patients, mean weekly dose 7,000 mg), hydroxychloroquine (79 patients, median weekly dose 1,402 mg), and non-steroidal anti-inflammatory drugs (Table 1). There were no differences in age, body mass index height, white blood cell or platelet counts, hemoglobin, serum creatinine, alanine aminotransferase, C-reactive protein, mean blood pressure, disease duration, or disease activity indicating that the physical conditions were comparable in the two groups (Table 1). Furthermore, there were no differences in the treatment duration or weekly dose of prednisolone, salazopyrin, hydroxychloroquine, or non-steroidal anti-inflammatory drugs, suggesting that the potential impacts on the biochemical parameters from other drugs were minimal. Doses and durations of medication used by the study subjects are shown in Table 1. The median weekly dose of MTX used in the subjects was 12.5 (mean ± SD = 10.3 ± 3.9 mg/week), which is slightly higher than that of our previous studies in the US [14,18,19].

**Table 1:** Demographic data of study subjects (n = 99)

Characteristics	Reference value/range	Non-MTX users (n = 32)	MTX users (n = 67)	***P *value**^**a**^
Male/female		8/24	10/57	

Age, years		53.0 (49.0 to 58.0)	53.0 (50.3 to 57.0)	0.979

BMI, kg/m^2^	18.5-23.9	22.8 (21.6 to 24.4)	21.8 (21.6 to 23.5)	0.725

WBC count, cells/mm^3^	5,000-10,000	6,400 (5,536 to 6,844)	5,900 (5,980 to 6,900)	0.745

Hb, g/dl	12-16	12.9 (11.7 to 13.2)	12.6 (12.2 to 12.9)	0.765

Serum creatinine, mg/dl	<1.4	0.90 (0.87 to 1.13)	0.80 (0.79 to 0.87)	0.222

ALT, IU/L	0-40	15.0 (15.0 to 29.1)	16.0 (15.3 to 20.0)	0.976

CRP (mg/dL)	<0.3	0.40 (0.53 to 1.65)	0.22 (0.52 to 1.75)	0.161

Systolic BP, mmHg	<140	130.0 (123.4 to 133.8)	129.0 (114.7 to 142.2)	0.634

Diastolic BP, mmHg	<90	80.0 (76.2 to 82.3)	79.0 (65.8 to 92.7)	0.732

Disease duration, years		14.1 (11.5 to 16.8)	12.0 (11.3 to 15.0)	0.456

Disease activity, HAQ		0.187 (0.11 to 0.80)	0.125 (0.20 to 0.49)	0.917

Physical activity, h/week		0.00 (0.37 to 3.85)	0.50 (1.14 to 2.41)	0.552

Methotrexate:				

Weekly dose, mg		NA	12.5 (10.5 to 12.5)	

Duration, months		NA	36.0 (45.8 to 77.8)	

Prednisolone users/total:		24/32 (75%)	64/67 (95.5%)	

Weekly dose, mg		43.7 (35.0 to 48.7)	35.0 (38.9 to 55.3)	0.167

Duration, months		34.0 (32.6 to 105.8)	48.0 (46.4 to 79.2)	0.180

Salazopyrin, users/total:		26/32 (81.25%)	58/67 (86.56)	

Weekly dose, mg		7,000 (7,882 to 11,233)	7,000 (9,051 to 11,825)	0.395

Duration, months		53.5 (38.8 to 105.1)	50.0 (51.5 to 86.4)	0.848

Hydroxychloroquine:		22/32 (68.75%)	57/67 (85.07%)	

Weekly dose, mg		1,404 (1,404 to 1,968)	1,400 (1,486 to 1,804)	0.241

Duration, months		11.5 (9.2 to 15.7)	13.0 (13.6 to 21.6)	0.644

Celebrex, users/total:		19/32 (59.37)	42/67 (62.68%)	

Weekly dose, mg		1,400 (1,581 to 2,250)	1,400 (1,561 to 2,005)	0.660

Duration, months		18.0 (14.0 to 30.8)	23 (-8.7 to 123.8)	0.829

Meloxicam, users/total:		10/32 (31.3%)	26/67 (38.8%)	

Weekly dose, mg		105 (78.6 to 110.3)	105 (78.8 to 98.8)	0.336

Duration, months		7.5 (0.78 to 23.6)	12.0 (9.6 to 27.4)	0.599

Results are expressed as median (95% CI) (n = 99).

^a^Data were analyzed by Mann-Whitney U test.

ALT = alanine aminotransferase; BMI = body mass index; BP = blood pressure; CRP = C-reactive protein; HAQ = Health Assessment Questionnaire; Hb = hemoglobin; WBC = white blood cells.

### Plasma lipid profiles in subjects with or without low-dose MTX treatments

Mean TC, TG, HDL-C, and LDL-C levels of the study subjects were within the normal range of healthy humans (TC <200 mg/dl; TG <150 mg/dl; HDL-C >40 mg/dl; LDL-C <130 g/dl). Among these subjects, 24.5% had elevated plasma TC, 6.1% had elevated TG, 5.1% had low HDL-C, and 13.2% had elevated LDL-C. No differences were detected in plasma TC, TG, HDL-C, LDL-C in non-MTX users and subjects taking MTX (MTX+) (Table 2). The associations among blood lipid profile indices were examined. Plasma TC correlated with TG (r = 0.36, p < 0.001, n = 99), with HDL-C (r = 0.387, p < 0.001, n = 99), and LDL-C (r = 0.874, p < 0.001, n = 99). Plasma TG concentrations correlated with LDL-C (r = 0.367, p < 0.001, n = 99) and inversely correlated with HDL-C (r = -0.306, p = 0.002, n = 99). The associations were also present in MTX(+) subjects and MTX(-) subjects when examined separately. The lipid profile also reflected the health status: BMI significantly related to TG (r = 0.397, p < 0.001, n = 99), LDL-C (r = 0.267, p = 0.007, n = 99) and inversely related to HDL-C (r = -0.227, p = 0.024, n = 99) concentrations. BMI significantly related to TC (r = 0.276, p = 0.024, n = 67), TG (r = 0.425, p < 0.001, n = 67), and LDL-C (r = 0.313, p = 0.010, n = 67) in the MTX(+) subjects. BMI inversely correlated with HDL-C (r = -0.383 p = 0.03, n = 32) in MTX(-) subjects.

**Table 2:** Blood lipid profiles and cholesterol metabolic gene expressions in study subjects

	Normal range	Non-MTX users (n = 32)	MTX users (n = 67)	***P *value**^**a**^
TC, mg/dl	≤200 mg/dl	167.5 (162.8 to 192.4)	172.0 (169.3 to 185.5)	0.367

TG, mg/dl	≤150 mg/dl	81.0 (75.7 to 105.1)	71.0 (73.2 to 90.7)	0.344

TC/HDL-C	<4	1.34 (1.25 to 1.71)	1.38 (1.30 to 1.59)	0.884

HDL-C, mg/dl	≥60 mg/dl	66.0 (59.4 to 71.8)	69.0 (65.0 to 74.0)	0.410

LDL-C, mg/dl	≤100 mg/dl	85.7 (79.6 to 102.6)	89.0 (85.8 to 99.9)	0.673

LDL-C/HDL-C		2.72 (2.51 to 3.07)	2.60 (2.53 to 2.88)	0.736

***ABCA/18S***		**0.09 (0.03 to 0.77)**	**0.15 (0.43 to 2.60)**	**0.021**

***HY27/18S***		**0.07 (-0.05 to 1.41)**	**0.19 (0.32 to 5.40)**	**0.037**

Results are expressed as median (95% CI) (n = 99). Normal ranges of plasma lipid profiles: TC (total cholesterol) ≤200 mg/dl; TG (triglyceride) ≤150 mg/dl; HDL-C (high-density lipoprotein-cholesterol) ≥60 mg/dl; LDL-C (low-density lipoprotein-cholesterol) ≤100 mg/dl.

^a^Data were analyzed by Mann-Whitney U test. Bold text in tables represent significantly different (p < 0.05)

ABCA1 = ATP-binding cassette transporter A1; HY27 = 27-hydroxylase; MTX = methotrexate.

### ABCA1 and HY27 expression in human PBMCs

**Figure 2:**
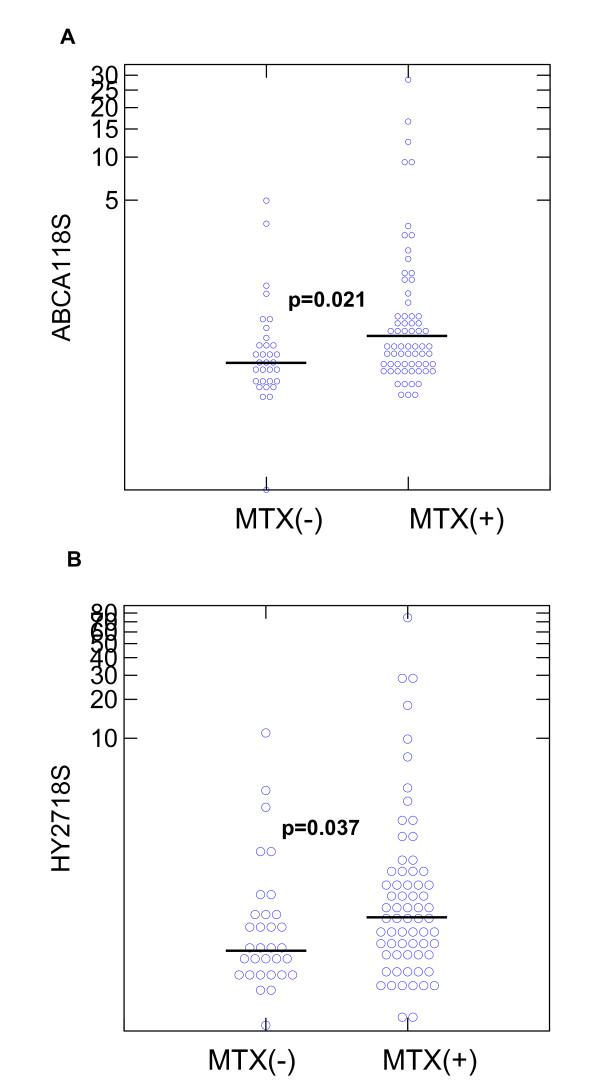
**Expressions of ATP-binding cassette transporter A1 (*ABCA1*) and 27-hydroxylase (*HY27*) in the peripheral blood mononuclear cells (PBMCs) of methotrexate (MTX)(-) and MTX(+) subjects**. **(a) **The distributions of PBMC *ABCA1 *expression in MTX(-) and MTX(+) subjects. **(b) **The distributions of PBMC *HY27 *expression in MTX(-) and MTX(+) subjects. The bars in the figures represent the median values of *ABCA1 *and *HY27 *in each study group.

Expressions of *ABCA1 *and *HY27 *were compared. The distributions of *ABCA1 *and *HY27 *gene expression in MTX(-) and MTX(+) subjects are shown in Figure 2a,b, respectively. In the PBMCs, MTX(+) subjects had significantly increased expressions of *ABCA1 *and *HY27 *mRNA compared to MTX(-) subjects (Mann-Whitney U test, p = 0.021 and 0.037 for *ABCA1 *and *HY27*, respectively).

No significant correlation was found between the MTX weekly dose or duration and the expression levels of *HY27*; however, an association between *ABCA1 *expression and the weekly dose of MTX was observed in MTX(+) subjects (r = 0.205, p = 0.042, n = 67). However, when using the median expression level as the cut-off for elevated gene expression, it was found that MTX(+) subjects were more likely to have elevated *HY27 *expression than MTX(-) subjects (OR = 2.5, p = 0.038) (Table 3). These results imply that *HY27 *expression in PBMCs might be induced by MTX treatment regardless of the dose or duration of MTX. The PBMC expression of ABCA tended to correlate with *HY27 *in all subjects (r = 0.172, p = 0.088, n = 99). In addition, ABCA expression tended to inversely correlate with HDL-C in MTX(+) subjects (r = -0.224, p = 0.068, n = 67).

**Table 3:** Stratified analysis between methotrexate (MTX) use and elevated ATP-binding cassette transporter A1 (ABCA1) and 27-hydroxylase expression in study subjects

Category			Logistic regression	
	
	Low (≤median)	High (>median)	OR (95% CI)	*P *value
*ABCA1/18S:*				

MTX				

No (n = 32)	20 (62.5%)	12 (37.5%)	1.00	

Yes (n = 67)	31 (46%)	36 (54%)	1.94 (0.82 to 4.58)	0.13

*27-Hydroxylase/18S:*				

MTX				

No (n = 32)	21 (66%)	11 (34%)	1.00	

Yes (n = 67)	29 (43%)	38 (57%)	**2.50 (1.04 to 6.01)**	**0.038**

Values represent N (%). Logistic regression results are expressed as OR (95% confidence interval) (n = 99).

### Dietary intake

Dietary intake of calories, fat, or cholesterol did not differ between MTX(-) and MTX(+) subjects (Table 4). Daily intake of energy (kcal/day), carbohydrate (g/day), protein (g/day), fats (g/day), and cholesterol (mg/day) were calculated and compared with the general population of the same age group. The percentage energy contributions from fats, protein, and carbohydrate were 28.6%, 13.3%, 58.1% in females and 25.5%, 13.5%, 61.0% in males, respectively. The percentage of energy contributions from fats, protein, and carbohydrates were 34.9%, 15.6% and 49.6%, respectively, in females or males between the ages of 35-54 [20,21]. In comparison with the general population of the same age group in the Nutrition and Health Survey in Taiwan (NAHSIT) [20-22], our subjects derived a lower percentage of energy from fats but more energy from carbohydrates (Table 4).

**Table 4:** Dietary intake in study subjects

Dietary intake	Reference value	Non-MTX users (n = 32)	MTX users (n = 67)	***P *value**^**a**^
Energy, kcal/day	2,000	1,248 (1,158 to 1,507)	1,249 (1,184 to 1,419)	0.862

Carbohydrate, g/day	300	183.5 (155.9 to 275.2)	174.5 (174.2 to 216.9)	0.994

Percentage of energy	60%	58.65%	55.94%	

Protein, g/day	(50)	51.1 (19.9 to 141.0)	38.9 (38.3 to 45.8)	0.849

Percentage of energy	10%	16.39%	12.48%	

Fat, g/day	(65)	43.9 (11.0 to 133.8)	38.6 (36.2 to 45.0)	0.505

Percentage of energy	<30%	31.68%	27.88%	

Cholesterol, mg/day	(<300)	243.8 (109.4 to 178.4)	223.6 (77.1 to 516.1)	0.493

Results are expressed as median (95%CI) (n = 99).

^a^Data were compared between methotrexate MTX- and MTX+ subjects by Mann-Whitney U test.

Most subjects (approximately 80%) of the present study had a daily cholesterol intake below 300 mg and only 7% had high fat intake (>65 g/day). These results indicated that our subjects had relatively healthy dietary habits, which may have contributed to the relatively normal blood lipid profiles in these subjects compared to those of the general healthy population [20-22]. The average time of physical activity (h) did not differ between the two groups.

## Discussion

This study demonstrated that clinical use of MTX for treating rheumatoid arthritis is associated with elevated expressions of atheroprotective protein HY27 and ABCA1 in human PBMCs. This is the first *in vivo *evidence in humans that the commonly used low-dose MTX in rheumatoid arthritis might induce the mRNA expression of antiatherogenic reverse cholesterol transporter expression in humans. Via the induction of these proteins, low-dose MTX treatment has the potential to protect against dyslipidemia through facilitation of cholesterol outflow in patients on this medication.

In the present study, no significant differences in blood lipids were observed between MTX(+) and MTX(-) subjects, possibly attributed to low prevalence of dyslipidemia in the study subjects. Although no differences were observed in the blood lipid profiles, the potential impacts of MTX on cholesterol metabolism should not be overlooked. The majority of our subjects had relatively normal cholesterol intake, and their lipid indicators fell within the normal range of healthy humans. We suggest that the potential impacts of altered *HY27 *and *ABCA1 *might be compensated by relatively normal cholesterol intake in these subjects. It is plausible that the potential atheroprotective effects from *HY27 *and *ABCA1 *inductions occur only in those persons with elevated blood lipids. This postulation needs to be investigated further in animals or subjects with abnormal blood lipids in the future.

According to the latest 2005-2008 NAHSIT, the prevalence of hyperlipidemia, hypertriglyceridemia, elevated LDL-C, and low HDL-C in females at the same age group were 4.7%, 11.8%, 18.6%, and 2.9%, respectively [22]. The prevalence of abnormal lipid profiles was determined by the standard for the threshold limit value of disease (TC ≥240 mg/dl, TG ≥200 mg/dl, HDL-C <35 mg/dl, LDL-C≥160 g/dl) [22]. When the same standard was applied to subjects in the present study, less than 5% of our subjects had elevated TC (4%) or TG (2%), elevated LDL-C (2%) or low HDL-C (2%), indicating that the prevalence of abnormal blood lipid profile in these subjects was indeed lower than that of the general Taiwanese population of the same age. When we looked at the prevalence of marginal elevated blood lipid profiles using the normal range of healthy humans (mean TC <200 mg/dl; TG <150 mg/dl; HDL-C >40 mg/dl; LDL-C <130 g/dl), the prevalence of dyslipidemia in our subjects was also lower than that in the NAHSIT [22]. Only 24.2% of our subjects had elevated TC, 6.1% had elevated TG, 5.1% had low HDL-C, and 12.1% had elevated LDL-C. Consistently, we found a lower prevalence of being overweight (24≤BMI <27 kg/m^2^) and obesity (≥27 kg/m^2^) in our study subjects (19% for being overweight and 16% for obesity) that was also lower than that of the general population at a similar age (39% for being overweight and 23% for obesity) in Taiwan [22].

The therapeutic strategies for dyslipidemia include a healthy diet with reduced intake of saturated fat and cholesterol. With regard to cholesterol intake, most of our subjects (79.6%) had a daily cholesterol intake below 300 mg (Table 4). In addition, only approximately one-fifth of these subjects had fat intake exceeding 30% of their total calories and, on average, 29% of the total energy came from fat. In the general population of the same age, the prevalence of high fat intake was up to 46.5%, and the energy from fats was 35% [22]. The mean intake of saturated fat in our study subjects was also significantly lower than that of the population [22]. We concluded that lower cholesterol and fat intake accounted for the relatively normal blood lipid profiles in these subjects.

*In vitro *MTX treatment was shown to increase *HY27 *mRNA expression [12] and reverse the COX2 inhibitor-induced downregulation of *HY27 *and *ABCA1 *in human THP-1 monocyte/macrophages [12]. Despite no differences being observed in the blood lipid profiles between MTX(-) and MTX(+) subjects, we found that clinical use of low-dose MTX was associated with increased *HY27 *and *ABCA1 *mRNA in the PBMCs. These results implied that under certain conditions, clinical use of MTX might induce the gene expression of atheroprotective protein *HY27 *and *ABCA1*. This is the first *in vivo *evidence that the commonly used DMARD MTX may increase the expression of antiatherogenic reverse cholesterol transport protein in human PBMCs. However, the potential impacts of MTX induced *HY27 *and *ABCA1 *expressions might be compensated by relatively normal to low cholesterol intake in these subjects; and that the potential atheroprotective effects from *HY27 *and *ABCA1 *may only be present in those persons with pre-existing dyslipidemia. Whether MTX induced *HY27 *and *ABCA1 *expressions can protect against cardiovascular disease in patients with chronic inflammation through the facilitation of cholesterol export remains to be established. Further studies pertaining to the potential benefits of low-dose MTX in hypercholesterolemic subjects are warranted.

## Conclusions

We demonstrated novel findings on the increased gene expressions of atheroprotective protein HY27 and ABCA1 in human PBMCs in subjects taking low-dose MTX for treating chronic inflammation. Although there is no direct evidence from this study that MTX may improve lipid profile, this is the first *in vivo *evidence in humans that the commonly used low-dose MTX in rheumatoid arthritis might induce the mRNA expressions of antiatherogenic reverse cholesterol transporters in humans. Via the induction of these proteins, low-dose MTX treatment has the potential to protect against dyslipidemia through facilitation of cholesterol outflow in patients on this medication. Further studies on the impacts of low-dose MTX on hypercholesterolemia are underway.

## Competing interests

The authors declare that they have no competing interests.

## Authors' contributions

All authors made substantive intellectual contributions to the present study and approved the final manuscript. E-PIC conceived of the study, generated the original hypothesis acquired funding, designed the study, performed data acquisition, statistical analysis, data interpretation, drafted and revised the manuscript. H-MC was in charge of clinical data acquisition,,statistical analysis, and literature review. D-YC and H-MC contributed equally on this work.D-YC and J-LL performing clinical assessments on study subjects. H-YC was involved with biochemical analyses. W-WC performed statistical analysis and dietary assessment.

## Author information

E-PIC, H-MC, H-YC, W-WC: Department of Food Science & Biotechnology, National Chung Hsing University, Taichung, Taiwan

H-MC:Department of Nursing and Pediatrics, Taichung Veterans General Hospital, Taichung, Taiwan

D-YC, J-LL: Division of Allergy Immunology Rheumatology, Taichung Veterans General Hospital, Taichung, Taiwan

D-YC, J-LL: National Yang-Ming University, Taipei, Taiwan

D-YC: Chung-Shan Medical University, Taichung, Taiwan

## Pre-publication history

The pre-publication history for this paper can be accessed here:

http://www.biomedcentral.com/1741-7015/9/4/prepub
